# Low-level expression of *Cmyc* in mature neurons: Maintaining neuronal function and preventing neurodegeneration

**DOI:** 10.4103/NRR.NRR-D-24-01367

**Published:** 2025-04-29

**Authors:** Qi Dong, Yanxia Ding, Yingxin Zhou, Xu Zhao, Lei Hu, Zhaohuan Zhang, Xiaohui Xu

**Affiliations:** 1 School of Preclinical Medicine, Wannan Medical College, Wuhu, Anhui Province, China; 2 Department of Laboratory Medicine, Changzheng Hospital, Naval Medical University, Shanghai, China; 3 Anhui Province Key Laboratory of Basic Research and Transformation of Age-related Diseases, Wannan Medical College, Wuhu, Anhui Province, China

**Keywords:** aging, c-Myc, dopaminergic neurons, Klotho, neurodegeneration, nitrated alpha-synuclein, Parkinson’s disease

## Abstract

*Cmyc*, a proto-oncogene, is expressed at extremely low levels in mature neurons and is traditionally thought to have no function in these cells. However, recent studies suggest that *Cmyc* may play a crucial role in maintaining the health and function of mature dopaminergic neurons. This study assessed the role of *Cmyc* in dopaminergic neurons and its significance in Parkinson’s disease. We used a conditional knockout approach to specifically delete *Cmyc* in substantia nigra dopaminergic neurons of adult mice. Our findings showed that *Cmyc* deletion led to progressive neuron loss, Parkinson’s disease-like symptoms, downregulation of Klotho, and upregulation of senescence-associated inflammatory factors, along with enhanced oxidative stress and nitrated alpha-synuclein accumulation, ultimately causing neuronal death. *In vitro* experiments confirmed increased senescence in *C-MYC* knockout cells, which was partially reversible by KLOTHO overexpression. We conclude that low-level *Cmyc* expression is essential for maintaining the health of mature dopaminergic neurons and preventing neurodegeneration, and suggest the c-Myc/Klotho axis as a potential therapeutic target for age-related neurodegenerative diseases, including Parkinson’s disease. Our study introduces a novel mouse model for Parkinson’s disease that replicates a condition associated with normal aging, offering a valuable tool for future research into disease mechanisms and therapeutic strategies.

## Graphical abstract

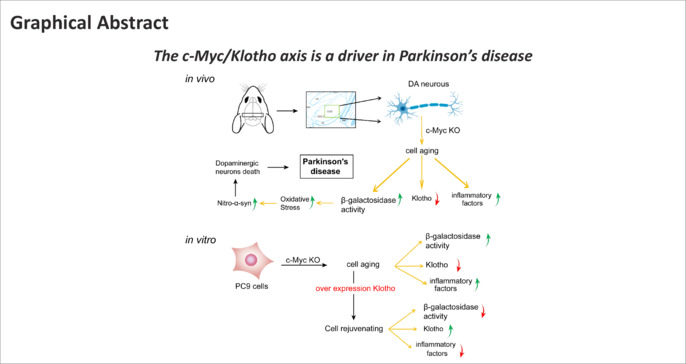

## Introduction

*C-MYC* is a proto-oncogene that has been extensively studied and is part of the MYC family, which includes *MYC* (*C-MYC*), *MYCN* (*N-MYC*), and *MYCL* (*L-MYC*) (Wang et al., 2021). The c-Myc protein regulates approximately 15% of human genes, including genes involved in cell cycle progression, metabolism, cell growth, differentiation, adhesion, and apoptosis (Boxer and Dang, 2001). Its influence extends beyond the nucleus, permeating the cellular microenvironment to orchestrate a diverse array of cellular processes (Gao et al., 2023). Moreover, *c-MYC* is a potent oncogene capable of driving unlimited cell proliferation, inducing immortalization, and stimulating cell division (Yoshida, 2018). Further, c-MYC is intricately linked to the division and proliferation of neural precursor cells during early nervous system development (Wey et al., 2010; Wang et al., 2020; Cai et al., 2021). When the *Cmyc* gene is knocked out during this early developmental stage, there is a significant reduction in the number of granular neurons, leading to major defects in brain development (Wey et al., 2010). However, the function of c-Myc in mature neurons and the adult nervous system is not fully understood. Early reports showed that c-Myc expression is extremely low in mature neurons, which do not divide, and is undetectable by in situ hybridization (Ruppert et al., 1986), leading to the belief that c-Myc is nonfunctional in nondividing and highly differentiated cells (Ferrer and Blanco, 2000). Furthermore, it has been observed that damage factors associated with spinal cord injury can lead to elevated c-Myc expression in neurons (Di Giovanni et al., 2003). This rapid and high expression of c-Myc is thought to induce neuronal death (Lee et al., 2011), though the mechanisms underlying this induced neuronal cell death remain to be elucidated.

Adult dopaminergic neurons play a crucial role in the central nervous system, particularly in the regulation of movement, motivation, and reward-related behaviors (Chen et al., 2024; Cui et al., 2025). Dysfunction or loss of these neurons is a hallmark of Parkinson’s disease (PD), where the degeneration of dopaminergic neurons in the substantia nigra leads to severe motor impairments (Becerra-Calixto et al., 2023; Ramesh and Arachchige, 2023; Rademacher and Nakamura, 2024). In the healthy adult brain, dopaminergic neurons maintain a relatively stable state, but various factors can disrupt their normal function and lead to neurodegeneration (Wen et al., 2025). These findings highlight the importance of understanding the mechanisms underlying dopaminergic neuron dysfunction to develop effective therapeutic strategies for neurodegenerative diseases.

Klotho, a single-pass transmembrane protein, has been increasingly recognized for its role in anti-aging and neuroprotection (Torbus-Paluszczak et al., 2018; Grillo et al., 2022). It has been shown to modulate multiple cellular pathways, including those related to oxidative stress, inflammation, and apoptosis (Kanbay et al., 2024). In the nervous system, Klotho expression is associated with the maintenance of neuronal integrity and function (Hosseini et al., 2024). Lower levels of Klotho have been implicated in neurodegenerative processes in several neurological disorders (Zhou et al., 2018; Castner et al., 2023).

Considering that high c-Myc expression is thought to induce neuronal cell death, we investigated whether the targeted disruption of the *Cmyc* gene in dopaminergic neurons could substantially mitigate neuronal cell death within a PD model, and thereby slow the disease progression.

## Methods

### Animals

*Cmyc*^flox/–^ heterozygous mice and wild type (WT) mice were procured from Shanghai Model Organisms Center in Shanghai, Inc. (License No. SCXK (Yu) 2020-0005). Heterozygous *Cmyc*^flox/–^ mice were crossed with C57BL/6 strain WT mice, and subsequent matings of the offspring were conducted to generate *Cmyc*^flox/flox^ mice. These mice were provided with *ad libitum* access to food and water during the study. The housing conditions were maintained at a room temperature of 20–25°C and humidity level of 40%–50%, with a 12-hour alternating light cycle with natural lighting and proper ventilation.

Adult male mice between 8 and 10 weeks of age, weighing 20–25 g, were randomly assigned to three groups: normal group (WT; *n* = 7), *Cmyc* knockout group (*Cmyc*-KO; *n* = 15), and knockout control group (Control-KO; *n* = 15). We used only male mice because PD is more prevalent in males than in females (Zirra et al., 2023). Both the *Cmyc* knockout and knockout control groups were further divided into three subgroups according to time points at 10, 30, and 60 days after the injection of the adeno-associated virus (AAV). Efforts were made to minimize the pain and discomfort in the experimental animals during the experiments. The animal protocols of this study were approved by the Ethics Committee of Wannan Medical College (approval No. WNMC-AWE-2024070) on March 6, 2024, and were conducted in accordance with the National Institutes of Health Guide for the Care and Use of Laboratory Animals (8^th^ ed., National Research Council, 2011). All experiments were designed and reported according to the Animal Research: Reporting of *In Vivo* Experiments (ARRIVE) guidelines (Percie du Sert et al., 2020).

### rAAV

Type 9 AAV PAAV-HSYN-MCHERRY-P2A-NLS-CRE-WPRE and control AAV PAAV-HSYN-MCHERRY-P2A-3xFlag-WPRE were purchased from GenScript Biotech (Shanghai, China).

### Gene identification

Mice genotypes were determined 3–4 weeks postpartum. DNA was extracted from mouse tails using a DNA extraction kit (Beyotime, Shanghai, China, Cat# D0063), according to the manufacturer’s protocol. The flox primer sequences used were: upstream, 5′-TGG GGC TTG GAC TGT AAG-3′ and downstream, 5′-AAG AAA AAC ACA AGT TGG CCC-3′. The polymerase chain reaction (PCR) mixture consisted of 15 μL: 4.5 μL of 2×Taq PCR Mix premix reagent (Tiangen, Beijing, China, Cat# Y1526), and 1 μL each of the upstream and downstream primers (10 μM). PCR amplification was performed using a Bio-Rad thermocycler (Bio-Rad, Hercules, CA, USA, C1000 Touch Thermal Cycler), with the following cycling parameters: initial denaturation at 94°C for 5 minutes, followed by 30 cycles at 94°C for 30 seconds, 58°C for 30 seconds, and 72°C for 30 seconds, with a final extension at 72°C for 7 minutes. After amplification, the PCR products were subjected to electrophoresis on a 1% agarose gel to separate the DNA fragments. Flox homozygous mice were then selected for further experiments based on the band positions: WT mice displayed one band at 356 bp, heterozygous mice showed two bands at 356 bp and 515 bp, and homozygous mice exhibited one band at 515 bp.

### Stereotactic brain injection

Mice were anesthetized by administering an intraperitoneal injection of 2% pentobarbital sodium (FWD Chem, Shanghai, China, Cat# 57-33-0) at a dosage of 45 mg/kg. Once anesthetized, the mice were secured on a stereotactic brain injector (Chengdu Taimeng Software Co., Ltd., Chengdu, China), and the knob was adjusted until the anterior and posterior fontanel points of the mouse brain were at the same level. Substantia nigra coordinates were determined using the mouse brain atlas (Paxinos and Franklin, 2019) and were relative to the Bregma point (+3.09 mm after the bregma, right front fontanelle +1.3 mm). AAV and control virus were injected into the right substantia nigra pars compacta (SNc) of mice at 4.5 mm under the dura mater. A volume of 1 μL was injected over a period of 5 minutes, followed by a 5-minute period before the needle was slowly withdrawn. The surgical site was then disinfected and sutured closed with a surgical needle. Postoperatively, the mice were kept on a heating pad for a week, and an equivalent amount of antibiotics was added to their daily drinking water to facilitate wound healing and enhance the postoperative survival rate.

### Open field test

The open field test was performed using the automatic analysis system from Chengdu Taimeng Software Co., Ltd. Mice were placed in the center of a 50 × 50 × 50 cm^3^ white, wooden open field box, which was divided into nine equal-area squares for subsequent behavioral analysis. Once the mouse was introduced into the apparatus, synchronized video timing was initiated to record the mouse’s activity over a 300-second period, during which the total distance traveled was measured. After each test, the experimental box was promptly cleaned of any mouse residue and deodorized with 75% ethanol to prevent olfactory interference with subsequent mice. The next mouse was recorded after the ethanol had fully evaporated.

### Tail suspension swing test

The mouse was held by the base of the tail, 4 cm from the root, at a height of 50 cm above the ground. We observed whether the mouse’s body tilted to the left or right, with the head moving at least 10 mm from either side of the vertical axis. After noting a tilt, the mouse was gently placed on the ground and observed for an additional 10 seconds before repeating the test. If the mouse did not tilt to either side within 30 seconds, it was placed on the ground to rest for 1 minute before repeating the test. In this experiment, manual recordings were taken for a total of 40 tilts.

### Terminal dUTP nick-end labeling analysis

Cell death was detected by a one-step terminal dUTP nick-end labeling (TUNEL) kit (Beyotime, Cat# c1068). Briefly, after the tissue sections were washed with phosphate-buffered saline (PBS) 2–3 times, the TUNEL detection solution containing TdT enzyme and fluorescent labeling solution was added to the sample and incubated in the dark at 37°C for 60 minutes. The sample was then washed and sealed with an anti-fluorescence quenching tablet (Biosharp, Hefei, China, Cat# BL739B), and the positive signal was directly observed by fluorescence microscope (Nikon, Tokyo, Japan, TS2R).

### Frozen slices

Mice were anesthetized by administering an intraperitoneal injection of 2% pentobarbital sodium at a dosage of 45 mg/kg. Following anesthesia, the left ventricle was rapidly perfused with 40 mL of ice-cold 0.9% NaCl solution at 4°C, followed by 30 mL of 4% paraformaldehyde solution at 4°C over a period of 10 minutes. The brain was then extracted. After fixation in 4% paraformaldehyde solution for 12 hours, the brain tissue was sectioned using a mouse brain slice mold with a coronal plane interval of 0.5 mm, and sections containing the substantia nigra and striatum were retained. These sections were subsequently dehydrated in sucrose solutions of 20% and 30% for 12 hours each. Once the tissue sank to the bottom, indicating sufficient dehydration, it was embedded in Optimal Cutting Temperature (Sakura, Japan Cat# 4583) compound. The substantia nigra and striatum were then frozen and sectioned into 25-µm-thick coronal slices using a freezing microtome (Thermo Fisher Scientific, Waltham, MA, USA, HM525NX).

### Immunofluorescence staining

The frozen tissue slices were thoroughly washed three times in PBS to eliminate any residual embedding agents and tissue preservatives. Membrane permeabilization was achieved using 0.1% Triton X-100 in a water bath at 37°C for 1.5 hours. Antigen retrieval was performed using citrate sodium buffer. After allowing the sections to naturally cool to room temperature, they were blocked with Beyotime sealing fluid (Cat# P0260) at 37°C for 1 hour. This was followed by the application of primary antibodies: rabbit anti-tyrosine hydroxylase (TH; 1:1000, Abcam, Cambridge, MA, USA, Cat# ab137869, RRID: AB_2801410), rabbit anti-dopamine transporter (DAT; 1:500, Abcam, Cat# ab184451, RRID: AB_2890225), rabbit anti-dopamine receptor D1 antibody (D1R; 1:500, Abcam, Cat# ab279713), mouse anti-dopamine receptor D2 (D2R; 1:100, Santa Cruz Biotechnology, Dallas, TX, USA, Cat# sc-5303, RRID: AB_668816), rabbit anti-Ki67 (1:200, Abcam, Cat# ab16667, RRID: AB_302459), and mouse anti-nitro-a/b-synuclein (1:200, Merck, Darmstadt, Germany, Cat# 36-012, RRID: AB_11212535), and incubation at 4°C overnight. The next day, the tissue sections were washed at room temperature, and then incubated with fluorescent secondary antibodies at 37°C for 2 hours: goat pAb to rabbit IgG (Alexa Fluor® 488) (1:1000, Abcam, Cat# ab150077) and goat pAb to Ms IgG (Alexa Fluor 555) (1:1000, Abcam, Cat# ab150114, RRID: AB_2687594), followed by incubation with 4′,6-diamidino-2-phenylindole (1:1000, Beyotime, Cat# c1002). Following the secondary antibody incubation, the sections were rinsed four times with PBS. Finally, the tissue was treated with an anti-fluorescence quencher (Biosharp, Cat# BL739B) and sealed with a coverslip. Images were captured using a Nikon inverted microscope (Nikon, TS2R).

### Analysis method of high-throughput sequencing

Brain tissue from the substantia nigra and striatum of mice (WT [*n* = 3], *Cmyc*-KO-30 days group [*n* = 3], Control-KO-30 days group [*n* = 3]) were collected for high-throughput sequencing analysis. After obtaining the differentially expressed genes (DEGs), we conducted Gene Ontology (GO) functional significance analysis and Kyoto Encyclopedia of Genes and Genomes (KEGG) Pathway significance analysis. First, we used the DESeq2 software (version:1.22.2) (http://www.bioconductor.org/packages/release/bioc/html/DESeq2.html) to normalize the gene counts in each sample, estimating gene expression levels using the BaseMean value and calculating the fold change. Subsequently, we performed differential expression analysis using the negative binomial test to assess the significance of differences (Love et al., 2014). Finally, we screened for differentially expressed protein-coding genes based on fold change and significance test results.

For the GO functional enrichment analysis, we counted the number of DEGs in each GO term and used the hypergeometric distribution algorithm to calculate the significance of enrichment for each GO term. The analysis results returned a *P*-value for enrichment significance (calculated using Fisher’s exact test for each term in Biological Process [BP], Cellular Component [CC], and Molecular Function [MF]). By integrating the results of the GO analysis with biological context, key genes for further study can be identified.

For the KEGG Pathway analysis, we annotated the differentially expressed protein-coding genes using the KEGG database and used the hypergeometric distribution test to calculate the significance of enrichment for each pathway term.

For the GSEA enrichment analysis, we calculated the enrichment scores for the differentially expressed genes, estimated the significance level of the enrichment score, and correct for multiple hypothesis testing. The default standard is a minimum gene set of 15 and a maximum gene set of 500.

### Western blotting

Proteins were extracted from the substantia nigra and striatum of mice using a Proteinase Inhibitor Cocktail (Merck, Cat# 04693132001) and RIPA Lysis Buffer (Beyotime, Cat# P0013B). Protein concentrations were determined using the BCA Protein Assay Kit (Biosharp, Cat# BL521A). Equal amounts of protein samples were resolved by 10% sodium dodecyl-sulfate polyacrylamide gel electrophoresis and then transferred to a polyvinylidene fluoride membrane (Millipore, Burlington, MA, USA) via the wet transfer method. After blocking with 5% bovine serum albumin, the membranes were incubated with the following primary antibodies: rabbit anti-TH (1:1000, Abcam, Cat# ab137869, RRID: AB_2801410), rabbit anti-DAT (1:500, Abcam, Cat# ab184451, RRID: AB_2890225), rabbit anti-D1R (1:1000, Abcam, Cat# ab279713), mouse anti-D2R (1:1000, Abcam, Cat# sc-5303, RRID: AB_668816), and mouse anti-glyceraldehyde 3-phosphate dehydrogenase (GAPDH; 1:10,000, ABclonal, Wuhan, China, Cat# AC033, RRID: AB_2769570), overnight at 4°C. The next day, the membranes were washed with tris-buffered saline with Tween 20 (TBST) for 10 minutes three times and then incubated with the corresponding secondary antibodies: HRP-conjugated goat anti-mouse IgG (1:10,000, ABclonal, Cat# AS003, RRID: AB_2769851) and HRP-conjugated goat anti-rabbit IgG (1:10,000, ABclonal, Cat# AS014, RRID: AB_2769854), for 2 hours at room temperature. After further washing with TBST for 10 minutes three times, the target protein bands on the membrane were developed using ECL chemiluminescence solution (Abbkine, Wuhan, China, Cat# BMU102), and the images were captured using a Bio-Rad imaging system (Bio-Rad, CFX Connect Real-Time System). The gray values of the target and reference bands were processed and analyzed using ImageJ software, with the relative protein expression quantified as the ratio of the target band’s gray value to that of the reference (GAPDH) band.

### Senescence-associated β-galactosidase staining

Senescence-associated β-galactosidase (SA-β-gal) staining was performed using a Cell Senescence β-Galactosidase Staining Kit (Beyotime, Cat# C0602). The PC9 cells and the substantia nigra and striatum tissue slices were washed 1–2 times with PBS, then β-galactosidase staining fixative was added for 15 minutes at room temperature. After the fixative was removed and the samples were washed with PBS 2–3 times, the staining working solution was added and incubated at 37°C overnight. The working solution was removed, and the samples were washed 1–2 times with 70% ethanol then placed under the microscope for observation (Nikon, TS2R).

### Quantitative reverse transcriptase-polymerase chain reaction

Total RNA was extracted from PC9 cells and the substantia nigra and striatum tissues using TRIzol reagent (Invitrogen, Carlsbad, CA, USA, Cat# 15596018CN). A total of 1 μg of RNA was reverse transcribed into cDNA using the Prime Script TMFAST RT reagent Kit (TaKaRa, Japan, Cat# RR047A). Then, 2 μL of cDNA was used for real-time fluorescence quantification of the RNA using 2× Universal SYBR qPCR mix (Biosharp, Cat# BL697A-1). Real-time fluorescence quantification was performed using a Bio-Rad thermocycler (Bio-Rad, CFX Connect Real-Time System), with the following cycling parameters: initial denaturation at 95°C for 5 minutes, followed by 40 cycles of 95°C for 10 seconds, 55°C for 30 seconds, and melt curve from 65°C to 95°C with 0.5°C increments for 5 seconds, with a final hold at 4°C. The specific primers are listed in **Additional Table 1**. To calculate the relative expression levels, the ΔCt method was used, where the Ct value of the target gene was subtracted from that of the reference gene (ΔCt = Ct(target) – Ct(GAPDH)). To compare gene expression between different experimental groups, the ΔΔCt method was used (ΔΔCt = ΔCt(experimental) – ΔCt(control)). The relative expression ratio was then calculated using the 2^–ΔΔCt^ method.

**Additional Table 1:** Primers used for quantitative reverse transcriptase-polymerase chain reaction

Target gene	Primer sequence (5’-3’)
hGAPDH	Forward: AGGACTCATGACCACAGTCCATGC
	Reverse: GATGACCTTGCCCACAGCCTT
hKLOTHO	Forward: GAAAAATGGCTTCCCTCCTT
	Reverse: ACAACTCCCCAAGCAAAGTC
hCCL5	Forward: CCAGCAGTCGTCTTTGTCAC
	Reverse: CTCTGGGTTGGCACACACTT
hCXCR4	Forward: ACTACACCGAGGAAATGGGCT
	Reverse: CCCACAATGCCAGTTAAGAAGA
hP21	Forward: TGTCCGTCAGAACCCATGC
	Reverse: AAAGTCGAAGTTCCATCGCTC
mGAPDH	Forward: AGGTCGGTGTGAACGGATTTG
	Reverse: GGGGTCGTTGATGGCAACA
mKLOTHO	Forward: TTGGGTCACTGGGTCAATCTC
	Reverse: CCGGCACGATAGGTCATGTT
mCCL5	Forward: GCTGCTTTGCCTACCTCTCC
	Reverse: TCGAGTGACAAACACGACTGC
mCXCR4	Forward: GAAGTGGGGTCTGGAGACTAT
	Reverse: TTGCCGACTATGCCAGTCAAG
mP21	Forward: CCTGGTGATGTCCGACCTG
	Reverse: CCATGAGCGCATCGCAATC

GAPDH: Glyceraldehyde 3-phosphate dehydrogenase; H: human; m: mouse.

### Knockout of the *c-MYC* gene in the PC9 cell line

The *c-MYC* knockout procedure in PC9 cells was conducted according to the methodology established in a previous study (Hu et al., 2023). Specifically, guide RNAs (gRNAs) designed to target the *c-MYC* gene were cloned into the lentiCRISPR v2 vector. Subsequently, a mixture of lentiCRISPR v2-sgCmycs (GentleGen, SuZhou, China), along with the packaging plasmids pMD2.G and pSPAX2, were transfected into 293FT cells at a ratio of 5:2:3. Lentivirus-containing supernatants were harvested from the transfected 293FT cells 48 and 72 hours after transfection and used to infect PC9 cells for an additional 24 hours. Cells successfully infected with the lentivirus were selected using puromycin (MedChemExpress, Monmouth Junction, MJ, USA, HY-B1743A) and further confirmed by western blot analysis.

### Overexpression of *Klotho* in the *c-MYC*-KO PC9 cells

We constructed two mouse Klotho overexpression plasmids, truncated KL and full-length KL, which were assigned to four groups: the *c-MYC*-KO group, the *c-MYC*-KO + empty vector group (transfected with empty vector plasmid), the *c-MYC*-KO + t-KL group (transfected with truncated KL plasmid), and the *c-MYC*-KO + KL group (transfected with full-length KL plasmid). Serum-free Dulbecco’s Modified Eagle Medium (DMEM; KeyGEN, Nanjing, China, Cat# KGL1211-500) was applied to *c-MYC*-KO PC9 cells 1 hour before transfection. Then, 2.5 μg of plasmid DNA was diluted with 100 µL of serum-free DMEM and mixed thoroughly to prepare the DNA dilution. Next, 2.5 µL of Neofect^TM^ DNA transfection reagent (Mayin, Beijing, China, Cat# TF201201) was directly added to the DNA dilution and gently mixed, then left at room temperature for 30 minutes. After replacing the serum-free DMEM with complete medium (fetal bovine serum, VivaCell, Shanghai, China, Cat# C04001-500), the transfection complex was added to the cell culture medium and gently mixed, then placed in the incubator for 3 days. The second transfection was performed after 3 days, and the culture was continued for another 4 days.

### Statistical analysis

Statistical analyses were performed using GraphPad Prism 9.0 software (version 9.5.0, for Windows, GraphPad Software, San Diego, CA, USA). One-way analysis of variance followed by Tukey’s *post hoc* test was used to analyze the data. Results are shown as the mean ± standard error of the mean. *P* < 0.05 was considered statistically significant.

## Results

### Hyperkinesia after *Cmyc* knockout in midbrain dopaminergic neurons

To investigate the role of *Cmyc* in mature midbrain dopaminergic neurons, we developed *Cmyc* conditional knockout mice (**Additional Figure 1A**). The *Cmyc* gene was selectively ablated in the right SNc dopaminergic neurons of these mice by stereotaxic injection of AAV (**Additional Figure 1B–D**). Sequencing analysis confirmed the successful knockout of the *Cmyc* gene in the right substantia nigra 3 weeks after AAV injection (**Additional Figure 1E**).

**Figure 1:**
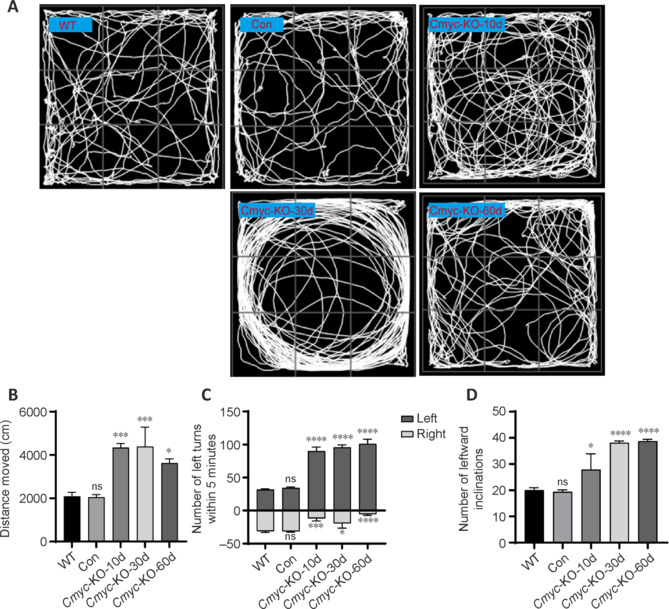
*Cmyc* knockout leads to behavioral changes in mice. (A) Open field test trajectory plots of the wild-type C57BL/6 group, control group, *Cymc*-knockout-10 days group (*Cmyc*-KO-10d) (*n* = 3), *Cmyc* knockout-30 days group (*Cmyc*-KO-30d) (*n* = 3), and *Cmyc* knockout-60 days group (*Cmyc*-KO-60d) (*n* = 3). All of the *Cmyc*-KO mice displayed curved movement trajectories accompanied by a leftward movement tendency. (B) The average total distance traveled of mice in the open field test. (C) The number of left turns within 5 minutes in the open field test. (D) The number of leftward inclinations in 40 iterations of the tail suspension swing test. Data are presented as the mean ± SEM. *P < 0.05, ***P < 0.001, ****P < 0.0001, *vs.* WT mice (one-way analysis of variance followed by Tukey’s *post hoc* test). KO: Knockout; WT: Wild-type.

Beginning 10 days after AAV administration, mice in the knockout group (*Cmyc*-KO) exhibited notable behavioral changes compared with WT mice and those in the control virus injection group. In the open field test, a 5-minute analysis of movement trajectories showed that *Cmyc*-KO mice displayed a curved movement pattern (**Figure 1A**), an inability to perform linear movements, a bias towards leftward movement, and an increased total distance traveled compared with WT and control mice (*P* < 0.05; **Figure 1B**). Statistical analysis of the frequency of left and right turns within a 5-minute interval showed that *Cmyc*-KO mice made significantly more left turns (*P* < 0.0001; **Figure 1C**) and significantly fewer right turns (*P* < 0.0001; **Figure 1C**) compared with WT and control mice.

In the tail suspension swing test, the number of leftward tendencies was recorded from 40 tests. The analysis indicated that *Cmyc*-KO mice showed a significant bias towards leftward movement compared with WT and control mice, starting from 10 days post-injection (*P* < 0.05; **Figure 1D**). This leftward turning tendency was stronger at later time points in the *Cmyc*-KO mice.

### Dysregulation of the substantia nigra-striatal neurotransmitter system after *Cmyc* knockout in midbrain dopamine neurons

**Figure 2:**
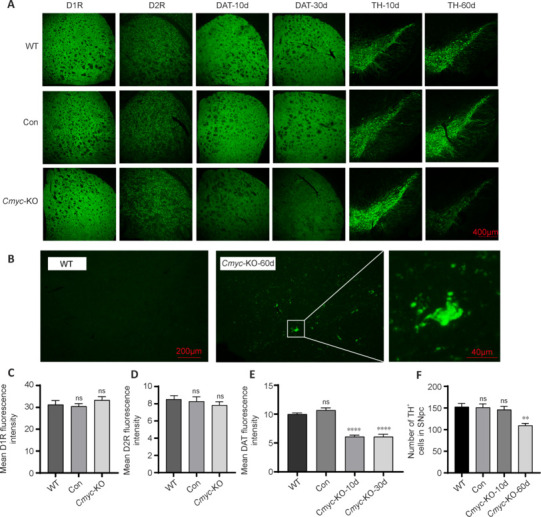
*Cmyc* knockout decreases the expression levels of DAT and TH in mice. (A) Immunofluorescence staining images of D1, D2, DAT in the striatum and TH in the substantia nigra pars compacta (SNpc) of WT, Control, and *Cmyc*-KO mice. From left to right showing green fluorescence signal: D1R, D2R, DAT and TH. The expression of DAT was decreased 10 days after *Cmyc* knockout (*n* = 3), and dopaminergic neurons were lost 60 days after *Cmyc* knockout (*n* = 3) in *Cmyc*-KO mice (scale bar: 400 μm). (B) TUNEL staining of WT mice and *Cmyc* knockout mice. Neuronal apoptosis occurred in the substantia nigra 60 days after *Cmyc* knockout. Scale bars: 200 μm for low magnification images and 40 μm for high magnification images. (C–E) D1R, D2R, and DAT fluorescence intensity. (F) The number of dopaminergic neurons (TH^+^ cells). **P < 0.01, ****P < 0.0001, *vs*. WT (one-way analysis of variance followed by Tukey’s *post hoc* test). Data are presented as the mean ± SEM. D1R: Dopamine D1 receptor; D2R: dopamine D2 receptor; DAT: dopamine transporter; KO: knockout; ns: not significant; TH: tyrosine hydroxylase; WT: wild-type.

**Figure 3:**
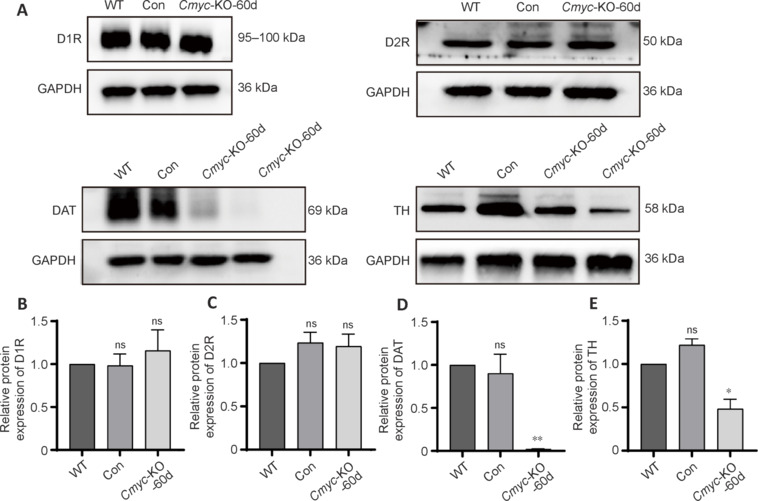
*Cmyc* knockout in midbrain dopamine neurons for 60 days decreases the expression levels of DAT and TH (western blot analysis). (A) Expression levels of D1R, D2R, DAT in the striatum and TH in the substantia nigra 60 days after *Cmyc* knockout in midbrain dopamine neurons. (B–E) Quantification of the expression levels of D1R (B) and D2R (C), DAT (D), and TH (E). *P < 0.05, **P < 0.01, *vs.* WT (one-way analysis of variance followed by Tukey’s *post hoc* test). Data are presented as the mean ± SEM (*n* = 3). D1R: Dopamine D1 receptor; D2R: dopamine D2 receptor; DAT: dopamine transporter; ns: not significant; KO: knockout; TH: tyrosine hydroxylase; WT: wild-type.

To investigate the underlying reasons for the observed behavioral changes, we focused on key markers within the dopaminergic neurotransmitter system, including D1R, D2R, DAT, and TH. Using immunofluorescence staining (**Figure 2**) and western blotting (**Figure 3**), we found that in *Cmyc*-KO mice, DAT expression was significantly reduced on the knockout side (right side) at 10 days post-knockout compared with both WT and control mice (*P* < 0.0001; **Figure 2E**). At this time point, no significant difference was observed in the number of dopaminergic neurons (*P* > 0.05; **Figure 2F**). After 30 days of *Cmyc* knockout in midbrain dopaminergic neurons, DAT expression in the striatum further decreased (*P* < 0.0001; **Figure 2E**), whereas TH expression remained unchanged (*P* > 0.05; **Additional Figure 2A**). At 60 days post-*Cmyc* knockout, DAT expression in the striatum continued to be reduced (*P* < 0.0001; **Additional Figure 2B**).

We then quantified the number of dopaminergic neurons in the SNc and found a significant reduction in *Cmyc*-KO mice (60 days) compared with that in WT mice (*P* < 0.01; **Figure 2F**). TUNEL staining indicated neuronal apoptosis associated with DNA double-strand break damage (**Figure 2B**), and Ki-67 staining showed no significant differences in cell proliferation between the three groups (**Additional Figure 2C**). Additionally, protein expression changes were assessed in striatal and SNc tissues 60 days after *Cmyc* gene knockout. We found decreased levels of DAT (*P* < 0.01; **Figure 3D**) and TH (*P* < 0.05; **Figure 3E**) in the *Cmyc*-KO group compared with WT and control mice.

### High-throughput sequencing analysis confirms the downregulation of *Klotho* expression and the activation of aging and inflammatory responses

**Figure 4:**
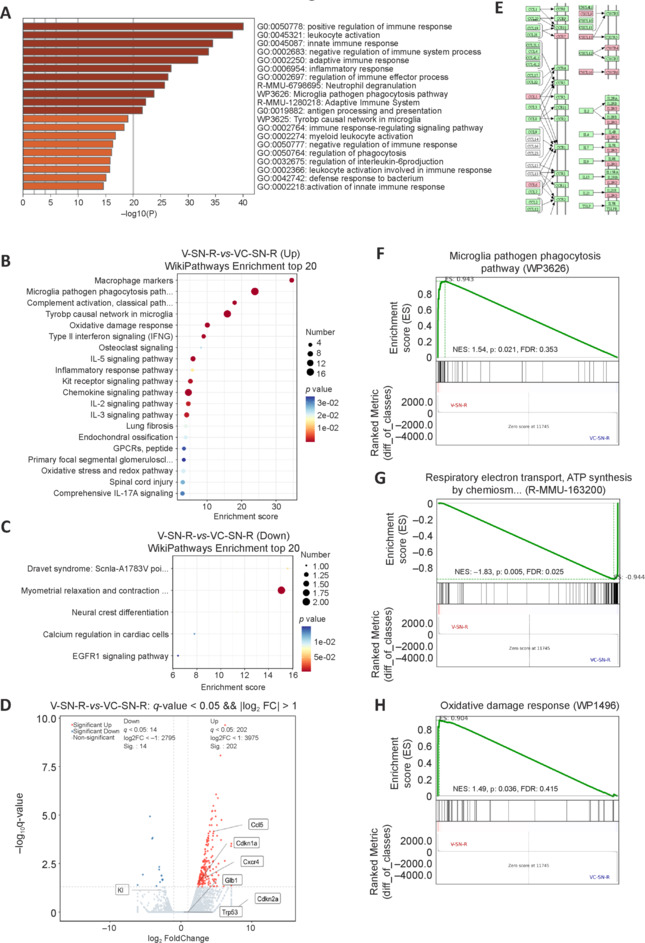
High-throughput sequencing results show that *Cmyc* knockout in midbrain dopaminergic neurons causes neuronal senescence. (A) Horizontal histogram displaying GO Enrichment top 20 pathways of upregulated DEGs of *Cmyc* KO groups compared with control groups. (B, C) Scatter plots of the top 20 KEGG enrichment of upregulated (B) and downregulated (C) DEGs of *Cmyc* KO groups compared with control groups. (D) Volcano plot displays DEGs of *Cmyc* KO groups compared with control groups. Significantly upregulated genes are colored red, significantly downregulated genes are colored blue. (E) The upregulated cytokines (red) of *Cmyc* KO groups in KEGG pathways. (F–H) Enrichment score of the microglia activation pathway (F), respiratory electron transport pathway (G), and oxidative damage response pathway (H) in *Cmyc* KO groups *versus* control groups analyzed by GSEA. *n* = 3. DEGs: Differentially expressed genes; GO: Gene Ontology; KEGG: Kyoto Encyclopedia of Genes and Genomes; KO: knockout.

**Figure 5:**
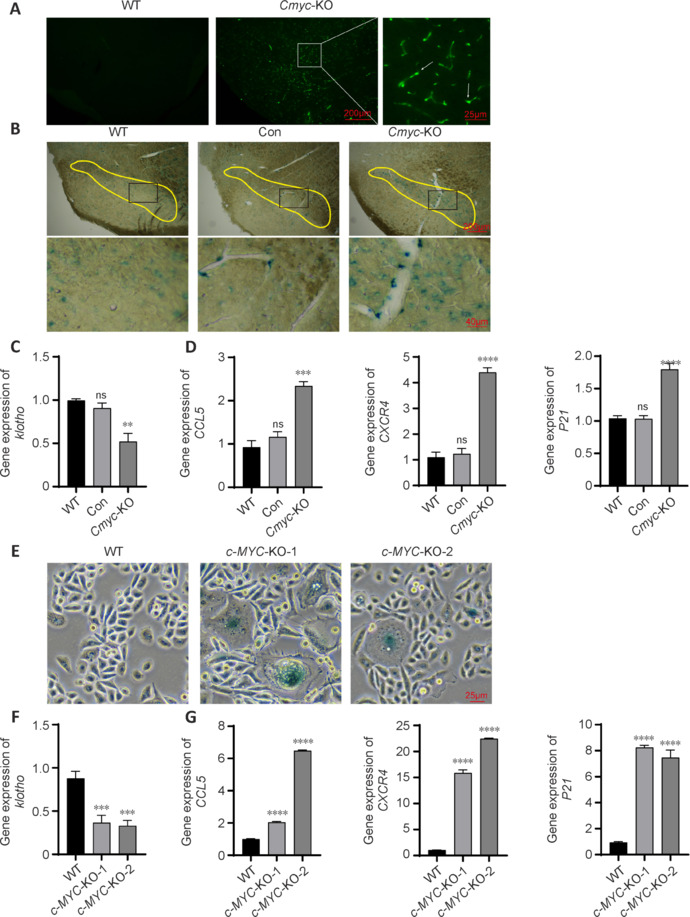
*Cmyc* knock out facilitates neuronal aging and nitro-α-syn aggregation. (A) At 60 days after *Cmyc* knockout (*n* = 3) in substantia nigra dopaminergic neurons, immunofluorescence staining in the substantia nigra region showed aggregation of nitro-α-syn. The green fluorescence indicated by the arrow is the neuraxond spheroid. Scale bars: 200 μm for low-magnification images and 25 μm for high-magnification images. (B) At 60 days after *Cmyc* knockout (*n* = 3), SA-β-gal staining in the substantia nigra region showed a deep blue product. Scale bars: 200 μm for low-magnification images and 40 μm for high-magnification images. Regions highlighted in yellow are the substantia nigra site. (C) *Klotho* expression in the substantia nigra region 60 days after *Cmyc* knockout (*n* = 3). (D) The mRNA expression of *CXCR4*, *CCL5*, and *P21* expression levels in the substantia nigra dopaminergic neurons 60 days after *Cmyc* knockout (*n* = 3). (E) In PC9 cells with *c-MYC* knockout, SA-β-gal staining showed a deep blue product compared with that in wild-type PC9 cells. Scale bar: 25 μm. (F, G) The mRNA expression of *klotho* (F) and *CCL5*, *P21*, and *CXCR4* (G) in PC9 cells after c-MYC knockout. Data are presented as the mean ± SEM. **P < 0.01, ***P < 0.001, ****P < 0.0001 (one-way analysis of variance followed by Tukey’s *post hoc* test). KO: Knockout; WT: wild-type.

To further investigate the effects of *Cmyc* knockout, we collected samples from the SNc of both control and *Cmyc*-KO groups for high-throughput sequencing analysis. Analysis of upregulated DEGs showed that the main non-redundant KEGG and GO terms enriched in the *Cmyc*-KO group were associated with immune and inflammatory responses (**Figure 4A** and **B**). Analysis of downregulated DEGs showed enrichment in pathways related to myometrial relaxation and contraction (**Figure 4C**). The volcano plot indicated 202 upregulated DEGs and 14 downregulated DEGs in the *Cmyc*-KO group compared with the control group. This analysis showed that *Cmyc* knockout in midbrain dopaminergic neurons led to significant downregulation of the anti-aging protein Klotho and upregulation of several aging markers, including GLB1, TP53, CDKN1A (p21), and CDKN2A (p16) (**Figure 4D**). **Figure 4E** illustrates the significant upregulation of cytokines related to aging (red) and their positions in KEGG signaling pathways. *Cmyc* knockout-induced aging in midbrain dopaminergic neurons also promoted microglia activation, reduced mitochondrial function, and increased oxidative stress in these neurons (**Figure 4F–H**). Additionally, immunofluorescence staining showed that nitro-α-syn was specifically present in the substantia nigra region of the *Cmyc*-KO group (**Figure 5A**).

β-galactosidase staining, a widely used senescence marker, showed that *Cmyc*-KO mice had an increased amount of dark blue staining products associated with senescence compared with that in WT and control mice (**Figure 5B**). Moreover, quantitative reverse transcriptase-polymerase chain reaction (qRT-PCR) results indicated significant downregulation of *Klotho* (*P* < 0.01; **Figure 5C**) and upregulation of *CCL5*, *CXCR4*, and *P21* (*P* < 0.001 or *P* < 0.0001; **Figure 5D**). β-galactosidase staining of *c-MYC*-knockout PC9 cells also showed an increase in the number of dark blue products compared with that in WT PC9 cells (**Figure 5E**). RT-PCR results showed significant downregulation of *KLOTHO* (*P* < 0.001; **Figure 5F**) and upregulation of *CCL5*, *CXCR4*, and *P21* (*P* < 0.0001; **Figure 5G**). These findings suggest that senescence phenotypes occur both in dopaminergic neurons of the mouse midbrain substantia nigra after *Cmyc* knockout and in the PC9 cell line after *c-MYC* knockout.

### Klotho overexpression in *c-MYC* knockout cells reduces senescence phenotype and inflammatory factors

Our high-throughput sequencing results showed that the downregulation of Klotho expression was associated with a cellular aging phenotype and a significant increase in inflammatory factors following *c-MYC* knockout. Given that Klotho is recognized as a pivotal anti-aging factor (Abraham and Li, 2022), and that its excessive administration can suppress the expression of inflammatory factors (Fan et al., 2022) and cell senescence (Liu et al., 2007; Abraham and Li, 2022), we aimed to determine whether Klotho overexpression could reverse *c-MYC* knockout-induced cell senescence.

**Figure 6:**
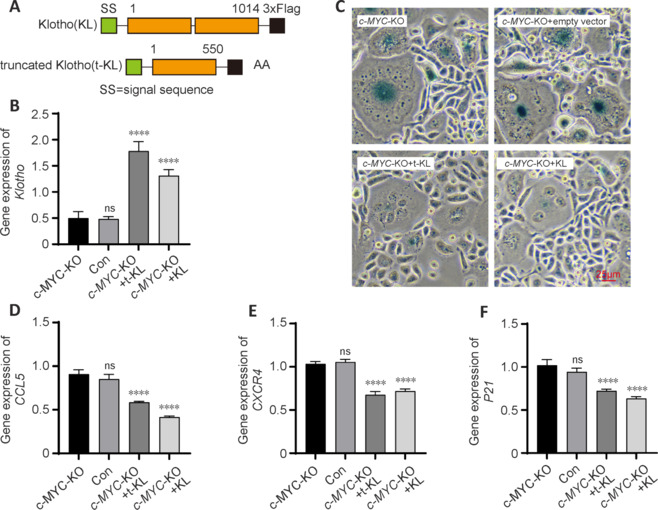
Klotho overexpression mitigates the aging phenotype and activation of inflammatory factors in *c-MYC* knockout cells. (A) Schematic diagram of the structures of the Klotho (KL) protein and truncated Klotho (t-KL) protein. (B) *Klotho* expression in c-MYC knockout cells after transduction of the klotho overexpression plasmid. (C) Deep blue product of SA-β-gal after klotho overexpression in c-MYC knockout cells. Scale bar: 25 μm. (D–F) The mRNA expression of *CCL5* (D), *CXCR4* (E), and *P21* (F) was decreased in *c-MYC* knockout cells after klotho overexpression. Data are presented as the mean ± SEM. ****P < 0.0001 (one-way analysis of variance followed by Tukey’s *post hoc* test). KO: Knockout; ns: not significant.

To investigate this, we constructed two mouse Klotho overexpression plasmids, truncated KL1 and full-length KL, and transfected them into PC9 cells to elevate *KLOTHO* expression. Schematic diagrams of the proteins expressed by these two plasmids are shown in **Figure 6A**. We assessed the expression levels of endogenous human *KLOTHO* in the *c-MYC* knockout group (c-MYC-KO), the group transfected with the empty vector (*c-MYC*-KO-empty vector), the group transfected with the truncated KL1 plasmid (*c-MYC*-KO+t-KL), and the group transfected with the full-length KL plasmid (*c-MYC*-KO+KL). We found that both the t-KL-KO and KL-KO groups had significantly increased endogenous Klotho expression compared with that in the c-MYC-KO group (*P* < 0.0001; **Figure 6B**). Moreover, SA-β-gal staining showed that overexpression of the *KLOTHO* gene in *c-MYC* knockout PC9 cells led to a reduction in β-galactosidase staining and a mitigation of cell senescence compared with the *c-MY*C-KO group (**Figure 6C**). RT-PCR results indicated that the expression levels of *CCL5* (*P* < 0.0001; **Figure 6D**), *CXCR4* (*P* < 0.0001; **Figure 6E**), and *P21* (*P* < 0.0001; **Figure 6F**) were decreased in both the t-KL-KO group and the KL-KO group compared with those in the *c-MYC*-KO group.

## Discussion

This study provides insights into the role of *c-MYC* in maintaining the youthful state of mature dopaminergic neurons and its implications in neurodegenerative diseases, particularly PD. The findings highlight the importance of low-level *Cmyc* expression in inhibiting neuronal senescence and preventing neurodegeneration. This study challenges the traditional view that c-Myc is not expressed or functional in mature neurons and indicates its role in maintaining neuronal health through the regulation of *KLOTHO* expression and inflammatory responses.

Our results are consistent with previous studies that have demonstrated the involvement of *Cmyc* in neurodevelopment and neural precursor cell proliferation (Ruppert et al., 1986; Wey et al., 2010; Wang et al., 2020). Additionally, our study extends this understanding by showing that low-level c-Myc expression in mature dopaminergic neurons is essential for maintaining their youthful state. This finding is in contrast with earlier studies that suggested c-Myc was not expressed in mature neurons (Ruppert et al., 1986; Ferrer and Blanco, 2000). The discrepancy may arise from differences in experimental approaches and the specific neuronal populations examined. Our conditional knockout model specifically targeted mature dopaminergic neurons, whereas previous studies focused on broader neuronal populations or developmental stages.

The significant downregulation of *Klotho* expression following *Cmyc* knockout aligns with studies indicating Klotho as an anti-aging factor (Liu et al., 2007; Abraham and Li, 2022). Further, our study demonstrates that c-Myc directly regulates *Klotho* expression in dopaminergic neurons, thereby influencing neuronal senescence and neuroinflammation. This regulatory relationship has not been previously described in the context of neurodegeneration. Additionally, our findings on the upregulation of senescence-associated inflammatory factors and oxidative stress markers after *Cmyc* knockout provide a mechanistic link between c-Myc, Klotho, and neurodegenerative processes.

Klotho can inhibit c-Myc expression (Lee et al., 2010), suggesting a negative feedback loop between these two factors. Our results further elucidate this relationship by demonstrating that *Cmyc* knockout leads to decreased Klotho expression, increased neuronal senescence and Increased oxidative damage can lead to nitro-α-syn aggregation in these neurons (Giasson et al., 2000). Our previous study showed that nitro-α-syn can directly kill dopaminergic neurons (Yu et al., 2010), which may also explain the death of dopaminergic neurons following *Cmyc* knockout. This feedback loop may play a crucial role in the progression of age-related neurodegenerative diseases such as PD. These findings highlight the importance of maintaining the balance between *Cmyc* and *Klotho* expression to prevent neuronal aging and death, which has not been emphasized in previous research.

Our study has some limitations. First, the use of a conditional knockout model in mice does not fully replicate the complexity of human neurodegenerative diseases. Although our model mimics PD caused by aging, the specific genetic and environmental factors contributing to human PD are not fully accounted for. Second, the study primarily focused on dopaminergic neurons in the substantia nigra, and the role of c-Myc in other neuronal populations remains to be explored. Third, the long-term effects of *Cmyc* knockout on neuronal function and behavior beyond the 60-day observation period were not determined. Finally, the study did not investigate the potential compensatory mechanisms that might occur in response to c-Myc deletion, which could influence the observed outcomes.

Future research should focus on exploring the detailed molecular mechanisms underlying the c-MYC/KLOTHO axis in dopaminergic neurons. Investigating the downstream targets of c-MYC and KLOTHO that mediate neuronal senescence and neuroinflammation could provide further insights into their roles in neurodegeneration. Additionally, studies should examine the potential therapeutic effects of manipulating c-Myc and Klotho expression in animal models of PD and other neurodegenerative diseases. Longitudinal studies tracking the progression of neuronal senescence and neurodegeneration over extended periods would also indicate the full impact of *Cmyc* knockout. Finally, exploring the role of c-Myc in other neuronal populations and comparing it with its function in dopaminergic neurons would provide a more comprehensive understanding of its role in the nervous system.

In conclusion, our study suggests that low-level c-Myc expression in mature neurons is important for maintaining neuronal function and preventing neurodegeneration. The loss of c-Myc in dopaminergic neurons results in reduced Klotho expression, increased inflammatory responses, and heightened oxidative stress, ultimately leading to neuronal loss and the manifestation of PD-like symptoms. Further, we showed that replenishing Klotho levels ameliorated the senescence associated with *Cmyc* deficiency. These findings suggest the therapeutic potential of targeting the c-MYC/Klotho axis to prevent age-related neurodegeneration, providing new avenues for the development of targeted therapies for PD.

## Additional files:

***Additional Figure 1:*** *Knockout of the Cmyc gene.*

Additional Figure 1 Knockout of the *Cmyc* gene.
(A) Identification of *Cmyc* homozygous mice. (B) Schematic for the generation of *Cmyc* knockout mice. (C, D) Immunofluorescent staining images of substantia nigra tissue sections after stereotactic injection of Dil-dye. Images show the successful injection of Dil dye into the substantia nigra site. (E) Sequencing results showed that a deletion of 2869 bp occurred between 1871 bp and 4740 bp of the *Cmyc* gene after injection of AAV virus, indicating that the *Cmyc* gene was indeed knocked out. AAV: Adeno-associated virus; TH: tyrosine hydroxylase.

***Additional Figure 2:*** *Dopamine system changes after Cmyc knockout for 30 and 60 days in the midbrain substantia nigra.*

Additional Figure 2 Dopamine system changes after *Cmyc* knockout for 30 and 60 days in the midbrain substantia nigra.
(A) Number of TH-positive neurons (green) at 30 days after *Cmyc* knockout. (B) Fluorescence intensity of DAT (green) in the striatum at 60 days after *Cmyc* knockout (green). (C) At 60 days after *Cmyc* knockout, no significant difference was detected in Ki-67 staining (green, Ki-67; blue, DAPI). Data are presented as the mean ± SEM. ****P < 0.0001 (one-way analysis of variance followed by Tukey's *post hoc* test). DAT: Dopamine transporter; DAPI: 4',6-diamidino-2-phenylindole; TH: tyrosine hydroxylase; WT: wil-type.

***Additional Table 1:*** *Primers used for quantitative reverse transcriptase-polymerase chain reaction.*

## Data Availability

*All relevant data are within the paper and its Additional files*.
