# Supplementary figures and images for: Low-level expression of Cmyc in mature neurons: Maintaining neuronal function and preventing neurodegeneration
Source: Neural Regen Res. 2025 Apr 29;21(6):2523–30. doi: 10.4103/NRR.NRR-D-24-01367 (PMC13211811; doi:10.4103/NRR.NRR-D-24-01367)

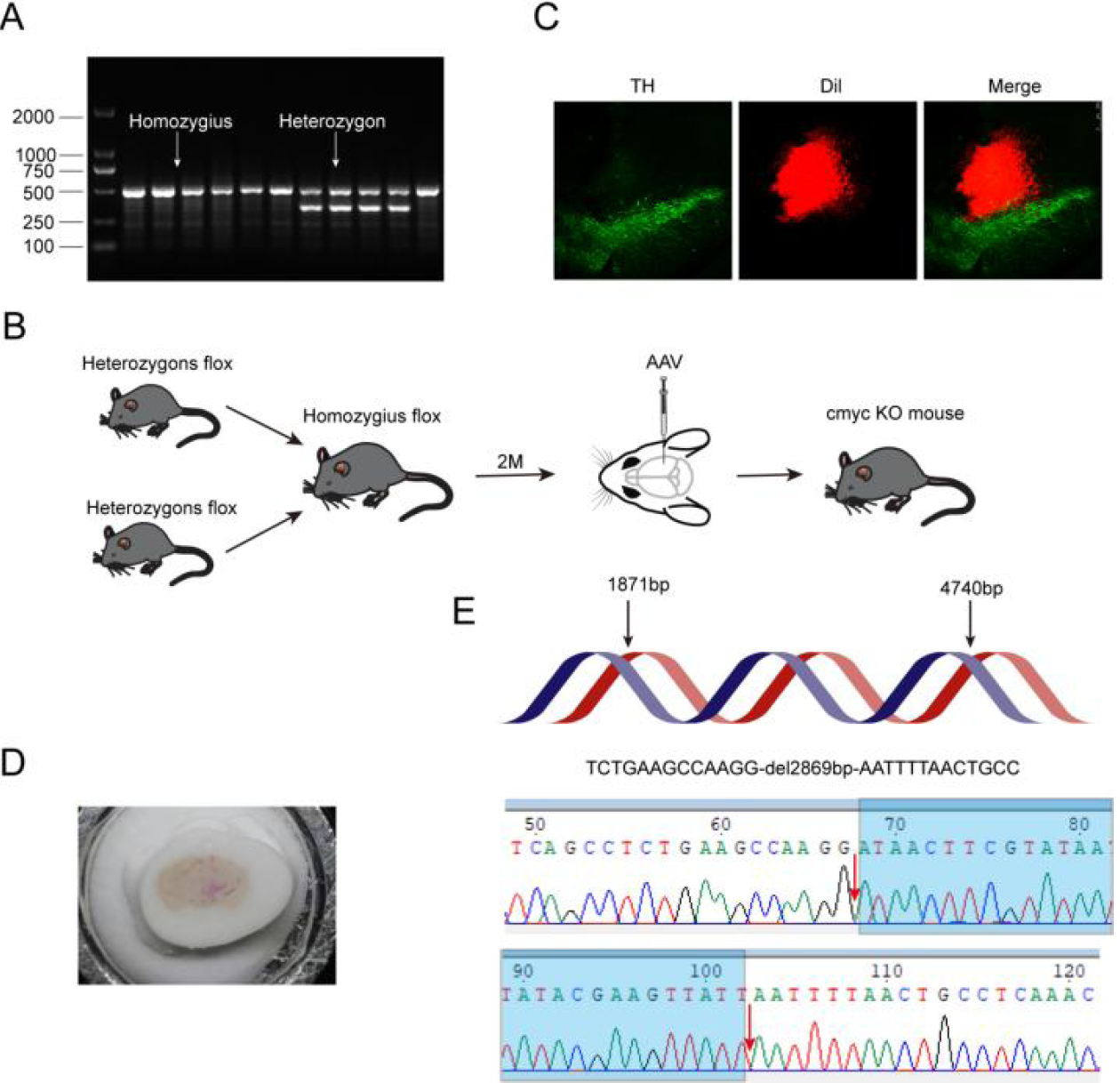

Supplement: Supplementary file 1 [file NRR-21-2523_Suppl1.tif]

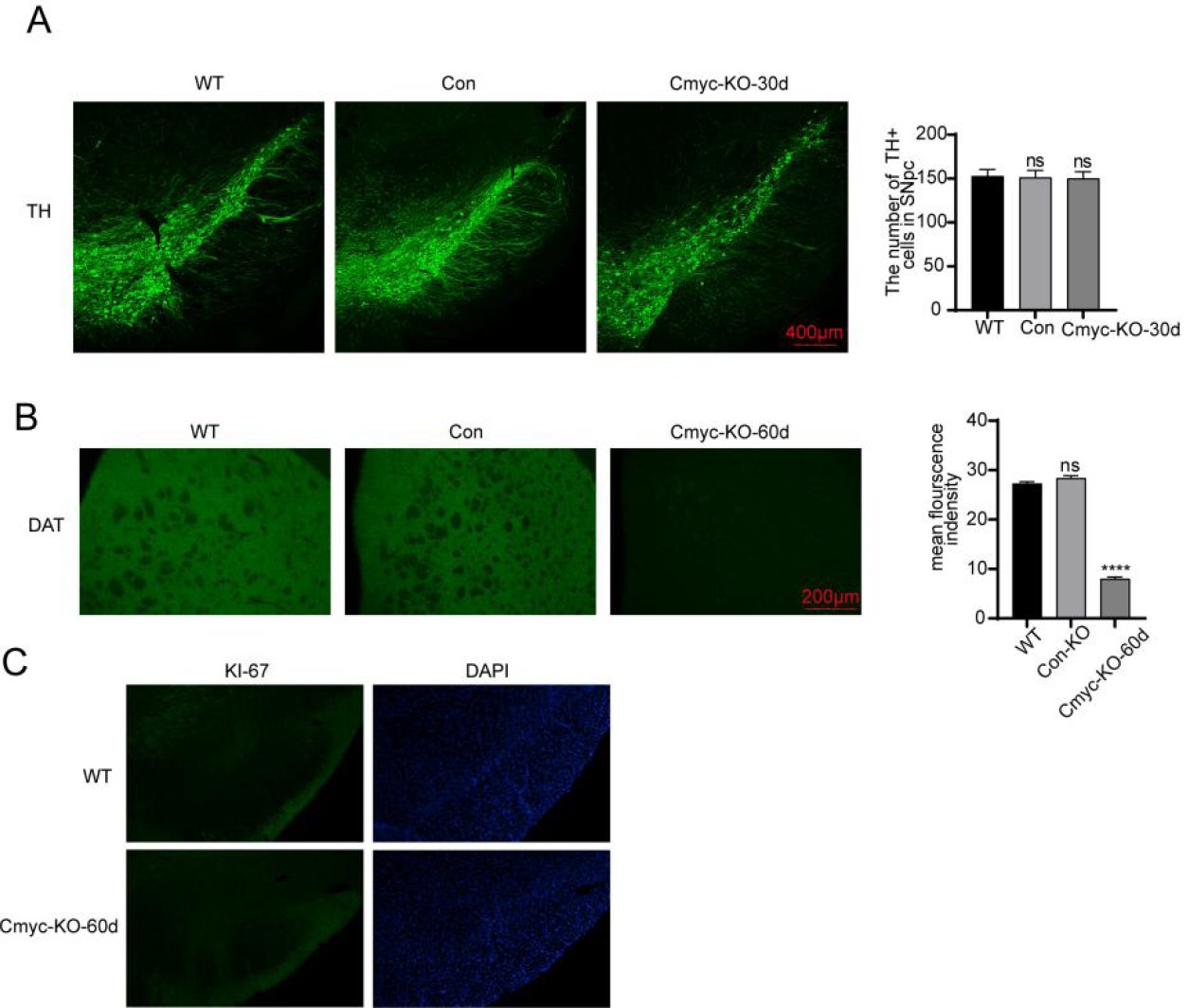

Supplement: Supplementary file 2 [file NRR-21-2523_Suppl2.tif]
